# Peripheral Nerve Conduction And Sympathetic Skin Response Are Reliable Methods to Detect Diabetic Cardiac Autonomic Neuropathy

**DOI:** 10.3389/fendo.2021.709114

**Published:** 2021-09-21

**Authors:** Xiaopu Lin, Chuna Chen, Yingshan Liu, Yu Peng, Zhenguo Chen, Haishan Huang, Lingling Xu

**Affiliations:** ^1^Department of Huiqiao Building, Nanfang Hospital, Southern Medical University, Guangzhou, China; ^2^Department of Endocrinology, Shenzhen Hospital, Southern Medical University, Shenzhen, China; ^3^The Third School of Clinical Medicine, Southern Medical University, Guangzhou, China; ^4^Department of Neurology, Nanfang Hospital, Southern Medical University, Guangzhou, China

**Keywords:** diabetic cardiac autonomic neuropathy, Ewing test, heart rate variability, nerve conduction, sympathetic skin response, T2DM

## Abstract

**Aim:**

This study aimed to investigate the role of nerve conduction studies (NCS) and sympathetic skin response (SSR) in evaluating diabetic cardiac autonomic neuropathy (DCAN).

**Methods:**

DCAN was diagnosed using the Ewing test combined with heart rate variability analysis. NCS and SSR were assessed by electrophysiological methods. The association between NCS/SSR and DCAN was assessed *via* multivariate regression and receiver-operating characteristic analyses.

**Results:**

The amplitude and conduction velocity of the motor/sensory nerve were found to be significantly lower in the DCAN+ group (all *P* < 0.05). A lower amplitude of peroneal nerve motor fiber was found to be associated with increased odds for DCAN (OR 2.77, *P* < 0.05). The SSR amplitude was lower while the SSR latency was longer in the DCAN+ group than in the DCAN– group. The receiver-operating characteristic analysis revealed that the optimal cutoff points of upper/lower limb amplitude of SSR to indicate DCAN were 1.40 mV (sensitivity, 61.9%; specificity, 66.3%, *P <* 0.001) and 0.85 mV (sensitivity, 66.7%; specificity, 68.5%, *P <* 0.001), respectively. The optimal cutoff points of upper/lower limb latency to indicate DCAN were 1.40 s (sensitivity, 61.9%; specificity, 62%, *P <* 0.05) and 1.81 s (sensitivity, 69.0%; specificity, 52.2%, *P <* 0.05), respectively.

**Conclusions:**

NCS and SSR are reliable methods to detect DCAN. Abnormality in the peroneal nerve (motor nerve) is crucial in predicting DCAN. SSR may help predict DCAN.

## Introduction

Diabetic cardiac autonomic neuropathy (DCAN) is a serious long-term complication of diabetes mellitus (DM). It results from chronic damage to autonomic nerve fibers that innervate the heart and blood vessels, in turn causing abnormalities in heart rate and vascular dynamics. It significantly increases the morbidity and mortality of patients with diabetes (1, 2). Early diagnosis of DCAN may reduce the risk of painless myocardial ischemia, myocardial infarction, and sudden cardiac death associated with DCAN (3). Therefore, the American Diabetes Association (ADA) recommends that the clinical screening for DCAN should be routinely performed in patients with diabetes (4).

Screening for DCAN is important because it is a frequently overlooked complication of diabetes, especially in the early stage. The Ewing test and heart rate variability (HRV) analysis are recommended by the ADA as the diagnostic tests for DCAN (5). The Ewing test, which assesses autonomic reflexes by measuring heart rate and blood pressure during activities such as the Valsalva, deep breathing, or standing, is a classic clinical test for cardiac autonomic neuropathy. However, it is a semi-quantitative method and relies on patients’ cooperation, and its interpretation is operator dependent. HRV analysis provides indirect insight into autonomic nervous system tone and plays a well-established role as a marker of cardiovascular risk (6).

HRV is composed of time-domain and frequency-domain components, but it does not estimate cardiac reflexes (7, 8). Some prior studies have demonstrated that the diagnostic specificity can be increased by combining the results of the Ewing test and HRV (5, 9), which was the approach used in this study.

Objective examinations that can identify DCAN during its early stage are critical. Considering that Ewing test and HRV analysis tests are time-consuming and require a high degree of cooperation from patients, it is difficult to screen for every diabetic patient in clinical work. Therefore, it is important to find a more convenient method to screen DCAN. Nerve conduction studies (NCS) and the sympathetic skin response (SSR) are routinely employed in the diagnosis of diabetic peripheral neuropathy (10, 11), which may be useful in identifying patients at high risk for DCAN. The present study was designed to investigate the role of NCS and SSR in the evaluation of DCAN in type 2 diabetes mellitus (T2DM).

## Materials and Methods

### Study Design

This cross-sectional, open-label, controlled clinical study was performed to explore the role of nerve conduction studies (NCS) and sympathetic skin response (SSR) in evaluating diabetic cardiac autonomic neuropathy (DCAN). Participants were recruited from the inpatients at the Department of Endocrinology of Nanfang Hospital. This study was reviewed and received ethical approval from the Ethics Committee of Nanfang Hospital, Southern Medical University. Written consent was obtained from all study participants at the time of enrollment. The Chinese Clinical Trials Registration Number is ChiCTR1900020491.

The inclusion criteria were as follows: age more than 18 years and current diagnosis of T2DM.

The exclusion criteria were as follows: inability to undertake the examination, neurological or autonomic disorders caused by other diseases (Guillain–Barre syndrome, Shy–Drager syndrome, and so forth), history of coronary artery disease (such as myocardial infarction or angina), arrhythmia, severe heart failure, malignant tumor, limb trauma, chronic rheumatic disease, thyroid function abnormalities, severe psychiatric disorders that interfered with the patient’s ability to complete study procedures, severe hepatic or renal dysfunction [GFR  <  30 mL/(min · 1.73 m^2^)], alcohol abuse (women 14 units per week and men 21 units per week in the last year), intake of neurotoxic medications or beta-blockers, vitamin B12 deficiency, and pregnancy.

The participants’ clinical data, including sex, age, duration of diabetes, height, body weight, body mass index (BMI), and smoking history, were recorded. Laboratory measurements, including triglyceride (TG), total cholesterol (TC), high-density lipoprotein cholesterol (HDL), low-density lipoprotein cholesterol (LDL), fasting plasma glucose (FPG), postprandial blood glucose (PBG), glycosylated hemoglobin (HbA1c), fasting C-peptide (FCP), and fasting insulin (FINS), were obtained after an 8-h fast.

### Ewing Test and HRV Analysis

The presence of DCAN was first assessed by measuring the four cardiovascular reflexes as described by Ewing in 1970: heart rate variation with deep breathing with an assessment of expiration to inspiration (E/I) ratio, heart rate analysis in the standing position (the 30 s/15 s ratio), Valsalva ratio, and blood pressure response to positional changes from lying to standing (orthostatic hypotension, OH) (12, 13). Three of these tests assessed the parasympathetic functions, such as E/I ratio, 30 s/15 s ratio, and Valsalva ratio, while OH assessed the sympathetic function (14) primarily. An E/I ratio more than the age-specific reference value, a Valsalva ratio ≥1.21, a posture ratio ≥1.04, and a systolic blood pressure reduction in response to the standing of ≤10 mm Hg were considered normal. An E/I ratio below the age-adjusted values, a Valsalva ratio ≤1.10, a posture ratio ≤1.00, and a systolic blood pressure fall in response to the standing of ≥20 mm Hg were considered abnormal. Values that fell between normal and abnormal were considered borderline. Each of these four tests was assigned a score of 0 for normal, 0.5 for borderline, and 1 for abnormal results; the sum of these four scores made up the Ewing score, which was used to assess the severity of DCAN. An Ewing score ≥2 was considered abnormal. Participants were instructed to avoid food and particular pharmacological agents (antidepressants, neuroleptics, nicotine, and caffeine) for 12 h preceding the examination.

HRV was evaluated according to the European Society of Cardiology guidelines (15) using a 24-h Holter monitor. Time-domain HRV and frequency-domain HRV indexes were analyzed. Time-domain HRV indexes included the standard deviation (SD) of all normal-to-normal (NN) intervals (SDNN), SD of the average NN intervals calculated over 5-min periods of the entire recording (SDANN), root mean square successive difference in the R-R interval (rMSSD, ms), and percentage of adjacent RR intervals with a difference of duration greater 50 ms (PNN50, %). The frequency-domain HRV indexes included low-frequency power (LF, ms^2^) and high-frequency power (HF, ms^2^). HRV was considered abnormal if at least two of the following six abnormal parameters were met: SDNN <50 ms, SDANN <40  ms, PNN50 <0.75%, rMSSD <15  ms, LF <300  ms^2^, and HF <300  ms^2^ (15, 16).

In this study, the presence of DCAN (DCAN+) was defined as having both an abnormal Ewing score (≥2) and an abnormal HRV analysis (≥2 abnormal parameters).

### Electrophysiologic Evaluation

NCS were performed using the Viking Quest (Nicolet VIASYS Healthcare, USA). Skin temperature was maintained above 32°C in the upper limbs and above 31°C in the lower limbs. The filtering frequency was 20 Hz to 10 kHz. The compound muscle action potential (CMAP) amplitude, latency, and motor conduction velocity (CV) of the median, ulnar, posterior tibial, and peroneal nerves were recorded. The CMAP amplitude was recorded from peak to peak by a supramaximal stimulation. The supramaximal stimulation was defined as 10% addition of stimulation charge after CMAP amplitude reaches its maximum. The measurements of sensory nerve action potential (SNAP) amplitude, latency, and sensory conduction velocity (CV) of the median, ulnar, and sural nerves were recorded also by supramaximal stimulation. The mean value of 20 results was taken for the sensory NCS. For each individual, the mean of the motor nerve amplitude was calculated using the following formula: Amplitude of motor nerve = (Amplitude of median nerve M + Amplitude of ulnar nerve M+ Amplitude of posterior tibial nerve M + Amplitude of peroneal nerve M)/4. The means of motor nerve CV, sensory nerve amplitude, and sensory nerve CV were calculated using the same method.

In the present study, diabetic peripheral neuropathy (DPN) was defined as the present of one or more abnormal nerve conduction result (amplitude or CV) in at least two different peripheral nerve (10).

### Sympathetic Skin Response

The SSR was studied using the standard method (17). The room temperature was maintained at 25°C–26°C. A standard electromyographic active electrode was attached to the right palm and sole and the reference electrode to the dorsum of the hand and foot. The stimulus used was a single electrical stimulus at the right wrist of 10 mA for 100ms duration. This stimulation procedure was standardized in previous studies on fibromyalgia and correlated with symptoms (18). Stimuli were delivered unexpectedly and at random intervals between 30 and 60 s. Five consecutive stimuli were delivered. The latency was measured from the onset of the stimulus artifact to the onset of the first negative deflection and expressed in seconds. The amplitude was measured from the baseline to the maximal negative peak and expressed in mV. The response was considered absent if no consistent voltage change occurred using a sensitivity of 50 mV per division after three trials at maximum stimulus intensity. Response latencies were considered pathological when exceeding two SD more than the mean latency in the control group. The SSR habituation was considered as the percent rate of the maximal amplitude change between the fifth and the first response. A value less than 1 indicated habituation.

### Statistical Analysis

For continuous variables, the results were presented as the mean ± SD if normally distributed, and median (interquartile range, IQR) if nonnormally distributed. The Student *t* test (normally distributed data) and the Kruskal–Wallis test (nonparametric data) were employed to examine differences between groups. Categorical data were analyzed using Pearson’s *χ* ^2^ test. Multivariate logistic regression was performed to determine which nerve parameter had the greatest predictive value for the diagnosis of DCAN. Receiver-operating characteristic (ROC) analysis was performed to assess the optimal SSR cutoff for indicating DCAN. A two-sided *P* < 0.05 was assumed to be statistically significant, while *P* < 0.001 was taken as highly significant. All statistical analyses were performed using SPSS statistics software (version 20.0, 2011; IBM, USA).

## Results

### Clinical Characteristics of Participants

From January 2019 to June 2019, 172 patients with T2DM were screened for inclusion in this study. Of these, 38 patients were excluded based on the listed exclusion criteria, and the remaining 134 participants were enrolled. Further, 42 patients (31.34%) were diagnosed with DCAN as described early (both positive Ewing test and positive HRV analysis). The average test results in each group are shown in **Table 1**. The baseline clinical and laboratory characteristics of the patients are also shown in **Table 1**. Patients with DCAN were more likely to be male compared with controls (*P <* 0.05), and were older (*P <* 0.001). Participants with DCAN also had a slightly lower FPG (*P <* 0.05). Diabetes duration, smoking, BMI, blood pressure, TG, TC, HDL, LDL, PBG, HbA1c, FCP, and FINS were similar between the two groups.

**Table 1:** Clinical characteristics of patients with T2DM and DCAN *versus* those without DCAN.

	DCAN+ (*n* = 42)	DCAN– (*n* = 92)	*P*
**Sex (M/F)**	30/12	48/44	<0.05^*^
**Age (year)**	63.86 ± 10.57	55.35 ± 12.63	<0.001^**^
**Duration (year)**	10.13 ± 7.59	8.25 ± 6.79	n.s.
**Smoking (%)**	26.5%	32.5%	n.s.
**BMI (kg/m^2^)**	24.22 ± 3.51	23.20 ± 3.09	n.s.
**SBP (mm Hg)**	148.38 ± 26.73	140.07 ± 24.11	n.s.
**DBP (mm Hg)**	81.95 ± 12.66	82.70 ± 11.45	n.s.
**TG (mmol/L)**	2.08 ± 1.83	1.82 ± 1.27	n.s.
**TC (mmol/L)**	4.55 ± 1.83	4.95 ± 1.57	n.s.
**HDL (mmol/L)**	0.97 ± 0.29	1.03 ± 0.28	n.s.
**LDL (mmol/L)**	2.85 ± 1.28	3.08 ± 0.99	n.s.
**FPG (mmol/L)**	8.45 ± 2.98	8.66 ± 3.34	<0.05*
**PBG (mmol/L)**	15.27 ± 7.30	14.97 ± 5.63	n.s.
**HbA1c(%)**	8.83 ± 2.74	9.62 ± 2.65	n.s.
**FCP (ng/mL)**	2.96 (1.07–3.69)	1.69 (1.02–2.73)	n.s.
**FINS (μU/mL)**	8.81 (3.60–15.44)	6.69 (3.49–13.79)	n.s.

Values were expressed as mean ± SD for normally distributed data and median with interquartile range for nonnormally distributed data, or n (%). Differences between the groups were analyzed using independent-sample t test for normally distributed values and using the Kruskal–Wallis test for nonparametric values. Pearson’s χ^2^ test was employed to analyze categorical data. BMI, Body mass index; SBP, systolic blood pressure; DBP, diastolic blood pressure; FCP, fasting C-peptide; FINS, fasting insulin; FPG, fasting plasma glucose; HDL, high-density lipoprotein; LDL, low-density lipoprotein; PBG, postprandial blood glucose; TG, triglycerides; TC, total cholesterol.

^*^P < 0.05, ^**^P < 0.001.

n.s., Not significant.

### Ewing Test Parameters Between DCAN+ and DCAN– Groups

Statistically significant differences were observed in E/I ratio (*P <* 0.001), Valsalva ratio (*P <* 0.001), 30 s/15 s ratio (*P <* 0.001), OH (*P <* 0.05), and Ewing score (*P <* 0.001) between DCAN+ group and DCAN– group (**Supplementary Table 1**).

### HRV Parameters Between DCAN+ and DCAN– Groups

Statistically significant differences were found in SDANN (*P <* 0.05), LF (*P <* 0.001), and HF (*P <* 0.001) between DCAN+ and DCAN– groups, while SDNN, rMSSD, and pNN50 were similar between the two groups (**Supplementary Table 2**).

### NCS Parameters Between DCAN+ and DCAN– Groups

There are 78.57% (33/42) patients diagnosed as DPN (based on NCS test) in DCAN group, while 59.78% (55/92) in non-DCAN group (*P* < 0.05). The averaged NCS results of different motor and sensory nerves were compared in **Table 2**. The amplitude (7.96 ± 2.66 mV *vs.* 9.60 ± 2.22 mV, *P <* 0.001) and CV (44.22 ± 5.10 m/s *vs.* 46.62 ± 3.96 m/s, *P <* 0.05) of the motor nerve were lower in the DCAN+ group than in the DCAN– group. Similarly, the sensory amplitude (17.90 ± 13.66 mV *vs.* 26.24 ± 11.92 mV, *P <* 0.001) and CV (48.13 ± 7.27 m/s *vs.* 51.16 ± 6.23 m/s, *P <* 0.05) were significantly lower in the DCAN+ group than in the DCAN– group (**Table 2**).

**Table 2:** Nerve conduction of patients with T2DM and DCAN *versus* those without DCAN.

		DCAN+ (*n* = 42)	DCAN– (*n* = 92)	*P*
**Motor nerve**	Amp (mV)	7.96 ± 2.66	9.60 ± 2.22	<0.001^**^
	CV (m/s)	44.22 ± 5.10	46.62 ± 3.96	<0.05^*^
**Sensory nerve**	Amp (mV)	17.90 ± 13.66	26.24 ± 11.92	<0.001^**^
	CV (m/s)	48.13 ± 7.27	51.16 ± 6.23	<0.05^*^

Data were presented as mean ± SD. Differences between the groups were analyzed using unpaired-sample t test.

Amp, Amplitude; CV, conduction velocity.

^*^P < 0.05, ^**^P < 0.001.

### Nerve Conduction for DCAN in Multivariate Logistic Regression

For logistic regression, the continuous variables were transformed into grade variables according to the reference value. Multivariate logistic regression analysis was performed with DCAN as dependent variables and the amplitude/CV of motor nerve for the median, ulnar, posterior tibial, and peroneal nerves as independent variables. Similarly, multivariate logistic regression analysis was performed with DCAN as dependent variables and the amplitude/CV of sensory nerve for the median, ulnar, and sural nerves as independent variables. As shown in **Table 3**, a lower amplitude of motor fiber of the peroneal nerve was found to demonstrate an increased risk for DCAN (OR 2.77, 95% CI 1.20–6.44, *P* < 0.05).

**Table 3:** Nerve conduction for DCAN in multivariate logistic regression.

Variables	Odds ratio (95% Cl)	*P* value
**Peroneal nerve M Amp**		
>2.6 mV	1 (Ref.)	
<2.6 mV	2.77 (1.20–6.44)	<0.05^*^

M, motor nerve; Amp, Amplitude.

^*^P < 0.05.

### SSR Value in DCAN+ and DCAN– Groups

The SSR amplitude of the upper limb [0.99 (0.16–2.70) mV *vs.* 2.18 (1.19–3.48) mV, *P <* 0.001] and lower limb [0.44 (0.13–1.38) mV *vs.* 1.17 (0.73–1.97) mV, *P <* 0.001] were lower in the DCAN+ group than in the DCAN– group. The SSR latency of the upper limb [1.49 (1.32–2.21) s*vs.* 1.36 (1.23–1.46) s, *P <* 0.05] and lower limb [1.99 (1.76–3.26) s *vs.* 1.79 (1.62–1.93) s, *P <* 0.001] were higher in the DCAN+ group than in the DCAN– group (**Table 4**).

**Table 4:** SSR of patients with T2DM and DCAN *versus* those without DCAN.

		DCAN+ (*n* = 42)	DCAN– (*n* = 92)	*P*
**Upper limb**	Amp (mV)	0.99 (0.16–2.70)	2.18 (1.19–3.48)	<0.001^**^
	Laten (s)	1.49 (1.32–2.21)	1.36 (1.23–1.46)	<0.05^*^
**Lower limb**	Amp (mV)	0.44 (0.13–1.38)	1.17 (0.73–1.97)	<0.001^**^
	Laten (s)	1.99 (1.76–3.26)	1.79 (1.62–1.93)	<0.05^*^

Amp, Amplitude; Laten, latency.

^*^P < 0.05, ^**^P < 0.001.

### ROC Analysis of SSR to Predict DCAN in Patients With T2DM

**Figure 1:**
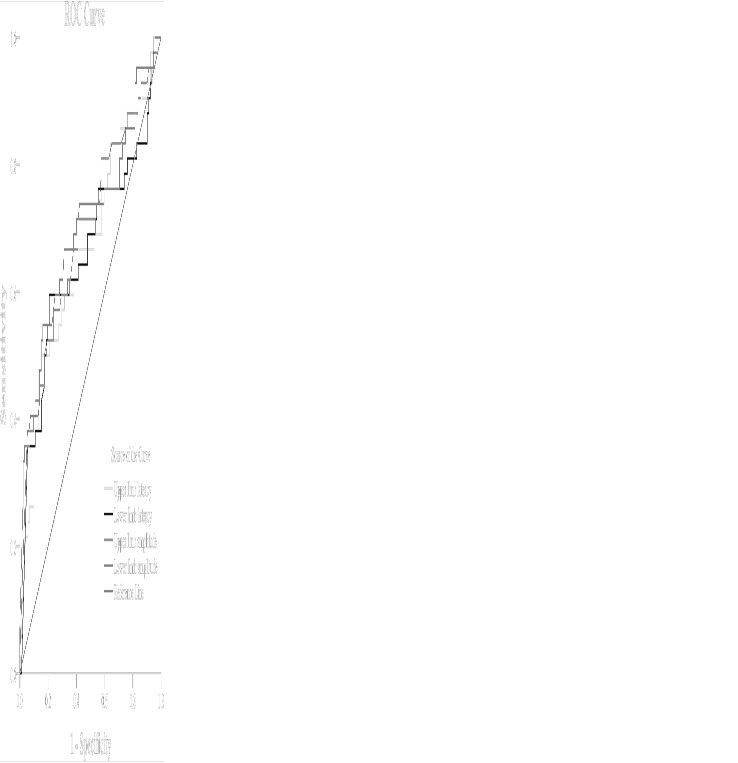
Receiver-operating characteristic (ROC) analysis of SSR to predict DCAN in patients with T2DM [(upper limb amplitude: AUC = 0.70; 95% CI: 0.59–0.80; sensitivity, 61.9%; specificity, 66.3%, cut-point 1.40 mV, *P* < 0.001) (lower limb amplitude: AUC = 0.70; 95% CI: 0.60–0.80; sensitivity, 66.7%; specificity, 68.5%, cut-point 0.85 mV, *P* < 0.001) (upper limb latency: AUC = 0.67; 95% CI: 0.57–0.77; sensitivity, 61.9%; specificity, 62%, cut-point 1.40 s, *P* < 0.05) [lower limb latency: AUC = 0.66; 95% CI: 0.55–0.77; sensitivity, 69.0%; specificity, 52.2%, cut-point 1.81 s, *P* < 0.05)].

ROC analysis revealed that the cutoff points of upper limb amplitude and lower limb amplitude, which maximized both the sensitivity and specificity to indicate DCAN, were 1.40 mV (AUC = 0.70; 95% CI: 0.59–0.80; sensitivity, 61.9%; specificity, 66.3%, *P <* 0.001) and 0.85 mV (AUC = 0.70; 95% CI: 0.60–0.80; sensitivity, 66.7%; specificity, 68.5%, *P <* 0.001), respectively. Similarly, the optimal cutoff points of upper limb latency and lower limb latency to indicate DCAN were 1.40 s (AUC = 0.67; 95% CI: 0.57–0.77; sensitivity, 61.9%; specificity, 62%, *P <* 0.05) and 1.81 s (AUC = 0.66; 95% CI: 0.55–0.77; sensitivity, 69.0%; specificity, 52.2%, *P <* 0.05), respectively (**Figure 1**).

## Discussion

DCAN results from damage to nerve fibers within the parasympathetic and sympathetic nervous systems. It is one of the leading causes of heart arrhythmias and is an independent risk factor for cardiovascular mortality among patients with diabetes. Early diagnosis and intervention in patients with T2DM can reduce DCAN progression. Recent guidelines strongly recommend screening for DCAN in patients with diabetes because the progression of cardiovascular denervation is partly reversible or can be slowed in the early stages of the disease (19).

In the present study, the overall prevalence of DCAN in a sample of patients with diabetes was 31.34%. The prevalence reported in previous studies ranged from 25% to 75% in patients with T2DM (1, 20, 21). The significant variability between studies might be attributed to the lack of uniform diagnostic criteria and underdiagnosis due to inadequate screening (1, 22). In this study, DCAN was diagnosed using the Ewing test combined with HRV analysis to increase diagnostic specificity.

The association between diabetes duration and the development of DCAN has not been established. Interestingly, in the present study, older age was associated with DCAN but diabetes duration was not. A recent study also suggested that the duration of DM had no significant association with HRV measurements (23). However, a previous review reported that the prevalence of DCAN in patients with T2DM was associated with increased age and duration of diabetes (24). Pop-Busui et al. showed that DCAN was associated with poor glycemic control, increased age, and duration of disease, and diastolic blood pressure (25). Most likely, DCAN started in the early stages of diabetes (26). The discrepancy in results between studies might be due to inconsistent DCAN diagnostic criteria.

In the present study, the Valsalva ratio, E/I ratio, 30 s/50s ratio, and OH were different between DCAN+ and DCAN− groups. Except for the rMSSD and PNN50, all the parameters of HRV analysis were lower in the DCAN+ group than in the DCAN− group, indicating that both parasympathetic and sympathetic dysfunction existed during the development of CAN in patients with diabetes. This conclusion was consistent with that of multiple previous studies (27, 28). The results of the present study extended and confirmed these findings and suggested that T2DM was a metabolic disease responsible for cardiac autonomic neuropathy due to damage to both sympathetic and parasympathetic fibers.

The proportion of DPN was higher in the DCAN+ group than that in the DCAN – group. This finding was consistent with previous research (29). The amplitude and CV of the motor and sensory nerves were lower in the DCAN+ group than in the DCAN– group. Patients with severe DCAN had significantly reduced nerve CV and amplitude of peripheral nerves (30); it appeared that patients with diabetes and DCAN suffered more frequently from peripheral neuropathy compared with those without DCAN (31). A positive association existed between low HRV during deep breathing and large nerve fiber neuropathy, the latter documented with electrophysiology and clinical examination (32). Although some studies did not agree with these findings (33), a possible explanation was that CAN diagnosis was not based on a unanimous definition.

The present study found that DCAN was closely related to impaired peripheral nerve conduction. Moreover, NCS abnormality in the peroneal nerve (motor nerve) was more important in predicting DCAN. This was in line with previous studies. For example, parallel involvement of peripheral neuropathy and DCAN may principally affect the more vulnerable large nerve fibers (34). Parallel development of DCAN and peripheral neuropathy is thought to involve vulnerable large nerve fibers, with recent evidence pointing to an association between parasympathetic nervous system impairment and reduced peroneal motor nerve CV (35).

Peripheral NCS examine large-fiber sensory and motor nerve conduction. However, SSR is a simple, noninvasive method for evaluating small-fiber sudomotor function (36, 37). The sudomotor function is primarily mediated by the stimulation of the post sympathetic cholinergic fibers, which reflect the sweat nerve glands (unmyelinated C fiber) function or density (38). SSR latency measurements reflect the function of unmyelinated C fibers; SSR amplitude measurements reflect the density of spontaneously activable sweat glands (39). SSR appears to be useful for assessing autonomic neuropathy in patients with diabetes (40). In the present study, both the latency and the amplitude of SSR were different between the DCAN+ and DCAN– groups. The ROC analysis in the present study indicated that patients with T2DM should be alert for DCAN if SSR had upper limb amplitude lower than 1.40 mV, lower limb amplitude lower than 0.85 mV, upper limb latency longer than 1.40 s, or lower limb latency longer than 1.81 s.

In the present study, the results suggested that a lower amplitude of peroneal nerve motor fiber, as well as a lower amplitude and longer latency of SSR, were associated with increased risk of DCAN. Therefore, NCS and SSR can be used to predict and assist in DCAN diagnosis Nevertheless, the small sample size of the study is a limitation. Prospective studies with a larger sample size and long-term follow-up are required to confirm this conclusion.

## Conclusions

The present study examined the role of NCS and sympathetic SSR in the evaluation of DCAN in diabetic patients. Consequently, this study revealed that a peroneal nerve (motor nerve) abnormality is crucial for predicting DCAN, wherein SSR may help. As DCAN is difficult to diagnose and predict, the data presented herein may be clinically useful.

## Data Availability Statement

The raw data supporting the conclusions of this article will be made available by the authors, without undue reservation.

## Ethics Statement

The studies involving human participants were reviewed and approved by Ethics Committee of Nanfang Hospital, Southern Medical University. The patients/participants provided their written informed consent to participate in this study.

## Author Contributions

Each author has made an important scientific contribution to the study and is thoroughly familiar with the primary data. XL and CC carried out the clinical studies, participated in the statistical analysis and drafted the manuscript. YL, YP, ZC, and HH carried out the data acquisition, participated in the manuscript preparation and literature research. LX conceived of the study, and participated in its design and helped to review the manuscript. All authors contributed to the article and approved the submitted version.

## Funding

This work was supported by Cultivating Plan Program for National Natural Science Foundation of Shenzhen Hospital, Southern Medical University under Grant NO. CNGZRJJPY202001.

## Conflict of Interest

The authors declare that the research was conducted in the absence of any commercial or financial relationships that could be construed as a potential conflict of interest.

## Publisher’s Note

All claims expressed in this article are solely those of the authors and do not necessarily represent those of their affiliated organizations, or those of the publisher, the editors and the reviewers. Any product that may be evaluated in this article, or claim that may be made by its manufacturer, is not guaranteed or endorsed by the publisher.
