# Supplementary material for: Peripheral Nerve Conduction And Sympathetic Skin Response Are Reliable Methods to Detect Diabetic Cardiac Autonomic Neuropathy
Source: Front Endocrinol (Lausanne). 2021 Sep 21;12:709114. doi: 10.3389/fendo.2021.709114 (PMC8490774; doi:10.3389/fendo.2021.709114)
Supplement: Supplementary file 1 [file DataSheet_1.docx]

**Supplementary data**

**Table 1. Ewing test of patients with T2DM and DCAN versus those without DCAN**

|  | **DCAN+ (*n* = 42)** | **DCAN– (*n* = 92)** | ***P*** |
| --- | --- | --- | --- |
| **E/I ratio**  **breaths/min)** | 7.75 ± 3.39 | 17.54 ± 7.23 | <0.001** |
| **Valsalva ratio** | 1.20 ± 0.16 | 1.44 ± 0.27 | <0.001** |
| **30 s/15 s ratio** | 0.99 ± 0.03 | 1.07 ± 0.13 | <0.001** |
| **OH (Δ mm Hg)** | 21.59 ± 17.73 | 13.18 ± 11.61 | <0.05* |
| **Ewing score** | 2.80 ± 0.53 | 0.96 ± 0.80 | <0.001** |

Data were presented as mean ± SD. Differences between the groups were analyzed using the unpaired-sample *t* test. E/I ratio, deep breathing with an assessment of expiration to inspiration; 30  s/15  s ratio, heart rate analysis in the standing position; OH, orthostatic hypotension.

^*^*P* < 0.05, ^**^*P* < 0.001.

**Supplementary data**

**Table 2. HRV of patients with T2DM and DCAN versus those without DCAN**

|  | **DCAN+ (*n* = 42)** | **DCAN– (*n* = 92)** | ***P*** |
| --- | --- | --- | --- |
| **Time domain** | | | |
| **SDNN (ms)** | 44.93 ± 21.41 | 51.67 ± 23.57 | <0.05^*^ |
| **SDANN (ms)** | 84.05 ± 35.65 | 101.59 ± 36.38 | <0.05^*^ |
| **rMSSD (ms)** | 39.50 (27.00–67.00) | 37.00 (24.00–58.5) | >0.05 |
| **pNN50 (%)** | 5.00 (2.00–9.00) | 5.00 (1.00–11.00) | >0.05 |
| **Frequency domain** | | | |
| **LF (ms^2^)** | 135.26 ± 104.33 | 326.87 ± 260.95 | <0.001^**^ |
| **HF (ms^2^)** | 64.35 (29.20–110.10) | 115.15 (61.70–231.60) | <0.001^**^ |

Data were presented as mean ± SD if normally distributed and median (interquartile range, IQR) if nonnormally distributed. The unpaired-sample *t* test (normally distributed data) and the Kruskal–Wallis test (nonparametric data) were used to examine differences among groups.

SDNN, SD of all normal-to-normal (NN) intervals; SDANN, SD of the average NN intervals calculated over 5-min periods of the entire recording; rMSSD, root mean square successive difference in the RR interval; PNN50, percentage of adjacent RR intervals with a difference in duration greater 50  ms; LF, low-frequency power; HF, high-frequency power.

^*^*P*<0.05, ^**^*P*<0.001.
